# Typing characteristics of metabolism-related genes in osteoporosis

**DOI:** 10.3389/fphar.2022.999157

**Published:** 2022-09-15

**Authors:** Jiandong Guo, Qinghua Huang, Yundong Zhou, Yining Xu, Chenyu Zong, Panyang Shen, Yan Ma, Jinxi Zhang, Yongfeng Cui, Liuqian Yu, Jiawei Gao, Gang Liu, Kangmao Huang, Wenbin Xu

**Affiliations:** ^1^ Hangzhou Ninth People’s Hospital, Hangzhou, China; ^2^ Zhejiang Provincial People’s Hospital, Hangzhou, China; ^3^ Shanghai Medical Innovation Fusion Biomedical Research Center, Shanghai, China; ^4^ Department of Orthopaedics, Sir Run Run Shaw Hospital, School of Medicine, Zhejiang University, Hangzhou, China; ^5^ Affiliated Hospital of Nantong University, Nantong, China

**Keywords:** osteoporosis, metabolic correlation, gene, typing features, biomarkers

## Abstract

**Objective:** Osteoporosis is a common musculoskeletal disease. Fractures caused by osteoporosis place a huge burden on global healthcare. At present, the mechanism of metabolic-related etiological heterogeneity of osteoporosis has not been explored, and no research has been conducted to analyze the metabolic-related phenotype of osteoporosis. This study aimed to identify different types of osteoporosis metabolic correlates associated with underlying pathogenesis by machine learning.

**Methods:** In this study, the gene expression profiles GSE56814 and GSE56815 of osteoporosis patients were downloaded from the GEO database, and unsupervised clustering analysis was used to identify osteoporosis metabolic gene subtypes and machine learning to screen osteoporosis metabolism-related characteristic genes. Meanwhile, multi-omics enrichment was performed using the online Proteomaps tool, and the results were validated using external datasets GSE35959 and GSE7429. Finally, the immune and stromal cell types of the signature genes were inferred by the xCell method.

**Results:** Based on unsupervised cluster analysis, osteoporosis metabolic genotyping can be divided into three distinct subtypes: lipid and steroid metabolism subtypes, glycolysis-related subtypes, and polysaccharide subtypes. In addition, machine learning SVM identified 10 potentially metabolically related genes, GPR31, GATM, DDB2, ARMCX1, RPS6, BTBD3, ADAMTSL4, COQ6, B3GNT2, and CD9.

**Conclusion:** Based on the clustering analysis of gene expression in patients with osteoporosis and machine learning, we identified different metabolism-related subtypes and characteristic genes of osteoporosis, which will help to provide new ideas for the metabolism-related pathogenesis of osteoporosis and provide a new direction for follow-up research.

## Introduction

Osteoporosis is a systemic metabolic bone disease with an increasing incidence rate. It is characterized by decreased bone mass, deterioration of bone microstructure, and increased risk of bone fragility and fracture (Author Anonymous, 1991). Osteoporosis is mainly characterized by osteoporotic fractures, including fractures in different body parts, especially hip fractures. The study predicts that 50% of women and 30% of men over 50 will suffer osteoporotic fractures (Johnell and Kanis, 2005). Osteoporosis exists in as many as 49 million people in North America, Europe, Japan, and Australia (Wade et al., 2014). Importantly, osteoporotic fractures lead to a reduced quality of life in different patients, including increased pain, morbidity, and mortality (Kerschan-Schindl et al., 2012; Hu et al., 2021). Studies have suggested that risk factors for osteoporosis include smoking (Bijelic et al., 2017), genetic and environmental factors (Bijelic et al., 2016), inadequate calcium and vitamin D intake, physical inactivity and inactivity, metabolic disorders, poor habits (smoking, excessive alcohol consumption, coffee), long-term treatment with corticosteroids, and presence of chronic diseases (Alibasic et al., 2013). However, in recent years, important progress has been made in the epidemiology, pathology, pathogenesis, early diagnosis, and treatment of osteoporosis. Regrettably, osteoporosis remains widespread worldwide. Osteoporotic fractures seriously jeopardize the quality of life and safety of older people, still cause high mortality and great medical burden, and the continuous increase in the number of patients deserves adequate attention and an appropriate multidisciplinary approach to prevention and treatment. Therefore, exploring the pathogenesis of osteoporosis phenotypic variation is key to improving current osteoporosis management and developing new therapeutic strategies.

Humans comprise a wide range of organisms, such as animals, that take up potential energy mainly through carbohydrates, fats, and proteins but also specific biochemical cascades involving several enzymes for catabolism. Physiologically, genes and pathways in many metabolic pathways are critical for many cellular metabolic functions. Therefore, dysregulation or imbalance of these genes and pathways can lead to cellular dysfunction and various metabolic diseases (Suzuki et al., 2020). Accumulating evidence suggests that metabolic factors play an important role in the pathogenesis of osteoporosis, which includes lipid metabolism (Alekos et al., 2020), glucose metabolism (Karner and Long, 2018; Cipriani et al., 2020), energy metabolism (Lee et al., 2017), and phospholipid metabolism (Palmieri et al., 2021), and metabolic syndrome (Chin et al., 2020).

Although there is substantial evidence for a possible link between bone disorders and metabolic disorders, the specific players and molecular interactions in these metabolic networks remain unclear. At the same time, omic technology has promoted the development and exploration of molecular changes in various clinical diseases. At the same time, using multi-omics technologies such as genomics, transcriptomics, proteomics, and metabolomics is the key to promoting the proper treatment of clinical diseases (Olivier et al., 2019). Therefore, it is of great significance to explore the mechanism of metabolic factors in osteoporosis. The development of specific genetic biomarkers for osteoporosis and biomarkers related to oxidative stress has been reported (Yang et al., 2019; Zhao et al., 2021a). Regrettably, no studies have reported the correlation between phenotyping characteristics of osteoporosis with metabolic genes. In this study, we collected osteoporosis microarray datasets from GEO to perform unsupervised clustering of metabolic gene expression according to gene expression for cluster analysis methods to reveal heterogeneity and classification among osteoporosis patients and identify metabolic key genes by machine learning. Subsequently, to identify osteoporosis subtypes by multi-omics analysis and trying at the level of biological information, diseases, pathways, and protein genes. Then, an independent dataset was used for validation. Finally, we explored representative immune and stromal cells for genes involved in the metabolism of osteoporosis, which may provide a new perspective on the pathogenesis of osteoporosis-related metabolic factor abnormalities.

## Materials and methods

### Data download

We identified candidate genes that contribute to gene expression from the Gene Expression Omnibus (GEO) databases downloaded from GeneChip with osteoporosis as a research study for subsequent analysis using “osteoporosis” and “Homo sapiens” as keywords, including GSE56814 (42 normal and 31 osteoporosis samples) (Zhou et al., 2018a), GSE56815 (40 normal and 30 osteoporosis samples) (Zhou et al., 2018b), GSE35959 (9 normal and five osteoporosis samples) (Benisch et al., 2012) and GSE7429 (10 normal and 10 osteoporosis samples) (Xiao et al., 2008). The data from gene chips GSE56814 and GSE56815 were combined using the sva function of the R language for subsequent analysis.

### Cluster analysis

Previous studies have identified 2,752 metabolism-related genes encoding all known human metabolic enzymes and transporters, which are available for download (Possemato et al., 2011). We used the ensemble similarity network fusion and consensus clustering algorithm (SNF-CC) to observe gene expression patterns and cluster identification in patients with osteoporosis (Wang et al., 2014). Before performing SNF-CC, a filtering procedure was first performed, including the exclusion of genes with low median absolute deviation (MAD) values (mad ≤0.5) in all patients with osteopetrosis. Then, the indices were clustered by cpi5 (cluster prediction index) and gaps statistics in the movies package of the R language. The most important parameter to estimate in any clustering study is the optimal number of clusters ķ For the data, where ķ is small enough to reduce noise but large enough to retain important information (Lu et al., 2020). Multi-omics heatmaps were drawn based on clustering results and used to identify, validate, and visualize molecular disease subtypes from multi-omics data.

### Differential gene expression profiles of metabolically related subtypes of osteoporosis

We used the R language limma package to perform gene comparison analysis between subtypes and the Venn diagram method to derive common representative DEGs. For example, to identify DEGs of subtype 1, draw a Venn diagram of (subtype 1 + subtype 2) v/s (subtype 1 + subtype 3) to obtain shared DEGs. The same method was used for the three subtypes, and *p* < 0.05 represented statistical significance.

### Pathway enrichment analysis and protein map visualization

Proteomics is a biomimetic visualization method for all bulk pathways of Homo sapiens generated by gene enrichment (Liebermeister et al., 2014). We visualize differential protein data in differential analysis using Proteomaps, a tool that displays the composition of the proteome with a focus on protein abundance and function. Each protein was displayed as polygonal tiles with one area representing protein abundance and functionally related proteins appearing in adjacent areas and used to observe dynamic changes in the proportion of Kyoto Encyclopedia of Genes and Genomes (KEGG) pathways between different patient groups.

### Screening of characteristic genes related to metabolism in osteoporosis

We constructed an SVM classifier using the top-ranked mrmr genes and applied an incremental feature selection (IFS) approach to determine the optimal number of genes as signature genes. The SVM function from package e10171 in the R language was used to implement the SVM method. We used to leave one out cross validation (loocv) to evaluate the predictive performance of each SVM classifier. Based on the LOOCV MCC for each candidate gene set, an IFS curve can be drawn. The x-axis represents the number of top genes used in the SVM classifier, and the y-axis represents the LOOCV MCC of the SVM classifier. According to the IFS curve, we can select an appropriate number of genes with good predictive performance as the final eigengenes (Cheng et al., 2020; Pei et al., 2020).

### Data validation of characteristic gene expression

We verified the expression of eigengenes and visualized the data by drawing violin plots. Gene chip GSE35959 and GSE7429 microarray data were used as external validation datasets.

### Immune correlates analysis

xCell is a novel method based on gene signatures that can infer 64 immune and stromal cell types (Aran et al., 2017). We visualized the relative enrichment of predetermined gene profile combinations via the immunedeconv function of the xcell package in the R language and performed association analyses based on gene expression data from 64 immune and stromal cell types (Aran et al., 2017). Correlation plot heatmaps were then drawn and visualized using ggplot2.

## Results

### Data download and analysis of different subtypes of osteoporosis

**FIGURE 1:**
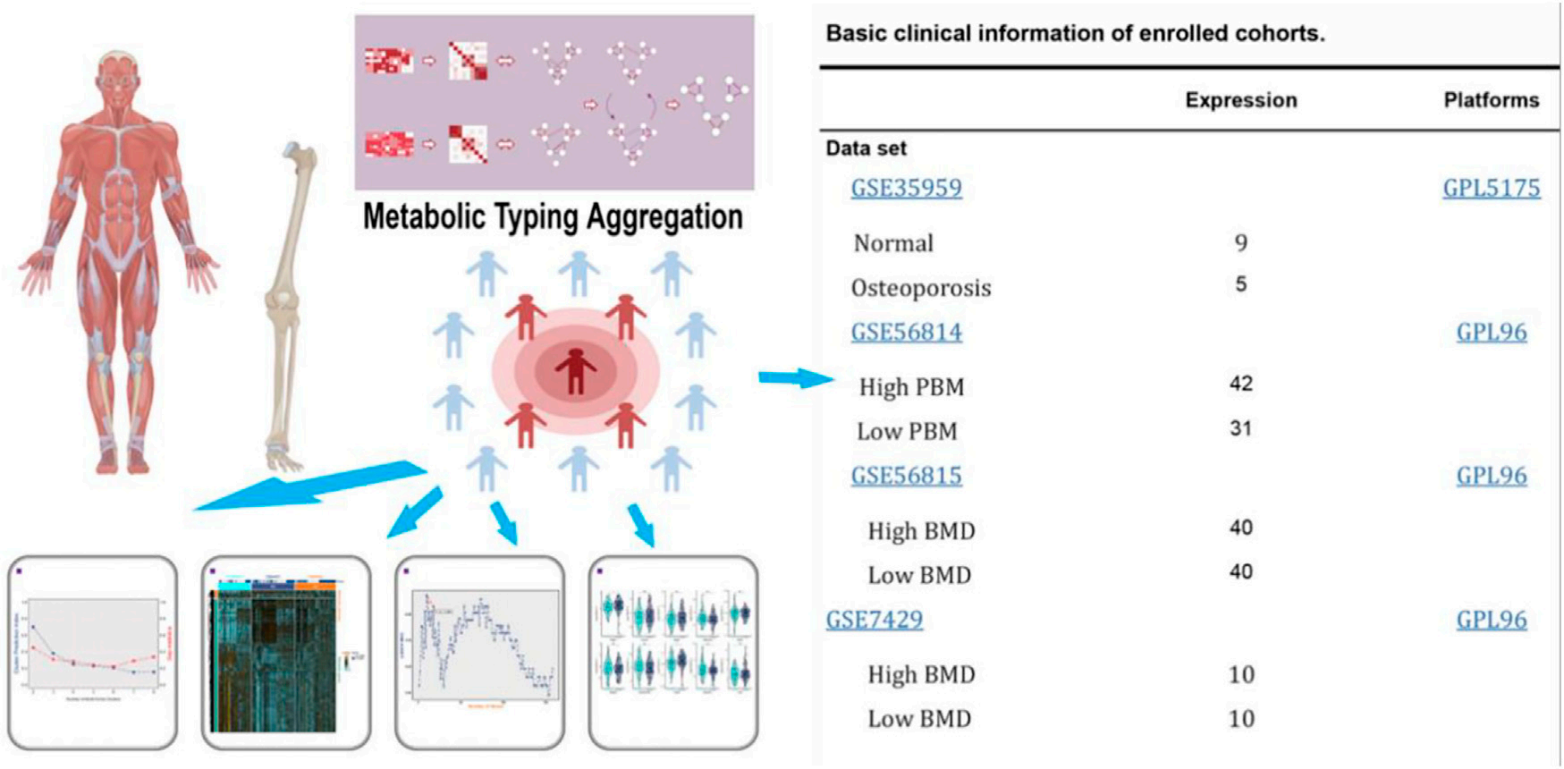
Flow diagram of the present study. The selected dataset contains the number of samples and cases.

**FIGURE 2:**
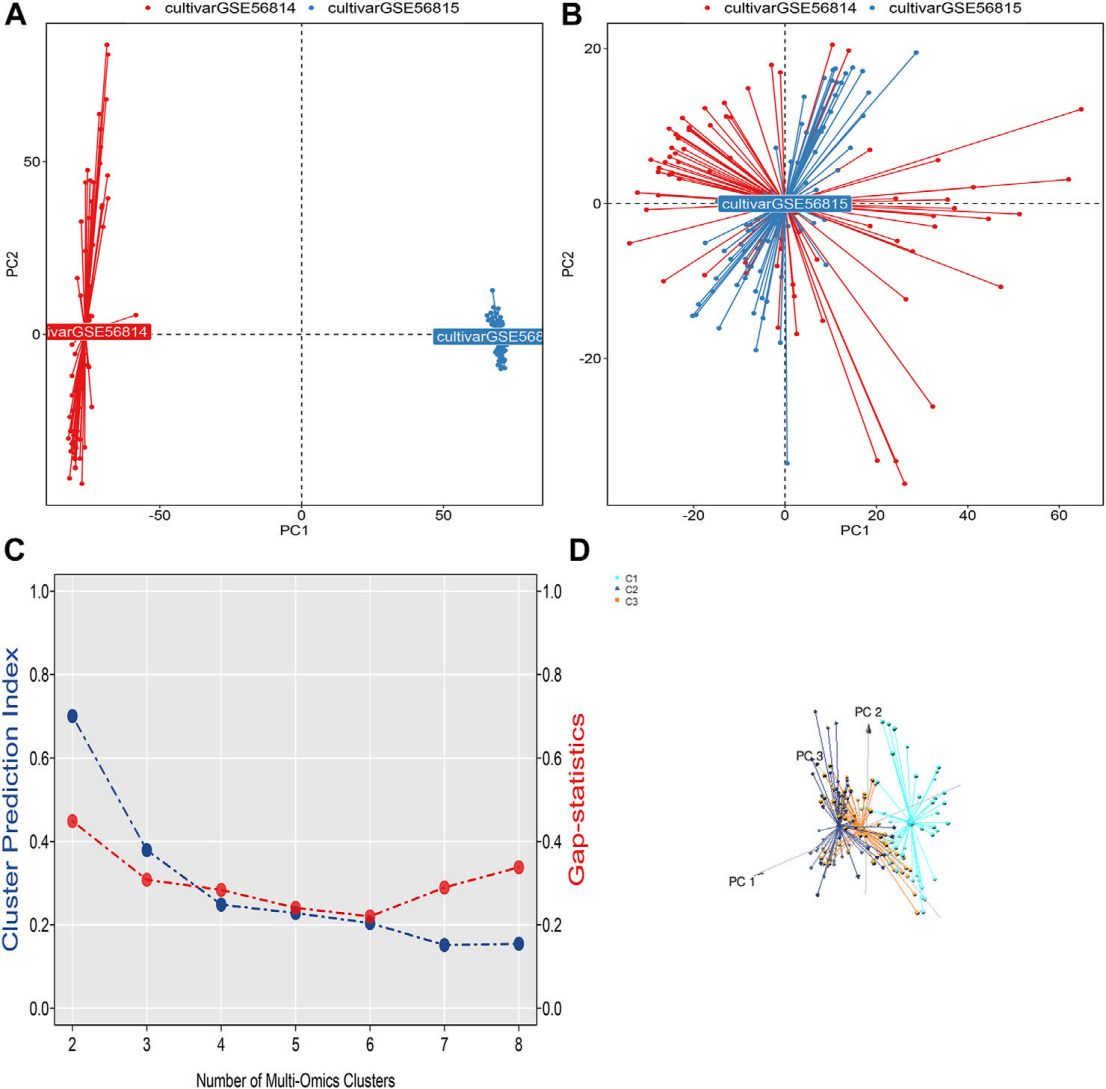
**(A)** Visualization of principal component analysis of expression profiles of GSE56814 and GSE56815 datasets. **(B)** Visualization of principal component analysis of the combined expression profiles of GSE56814 and GSE56815. **(C)** The optimal number of clusters k = 3 was identified by calculating the CPI (blue line) and gap statistic (red line) in the osteoporosis patient cohort, and the amount of gene expression in osteoporosis patients were classified into three different subtypes by ensemble similarity network fusion and consensus clustering algorithm (SNF-CC): C1, C2, and C3. **(D)** Visualization of principal component analysis of three subtype expression profiles.

The flow chart of this study is shown in Figure 1. We downloaded gene expression and clinical data of osteoporosis patients from the Gene Expression Omnibus (GEO) database, including GeneChip GSE56814, GSE56815, GSE35959, and GSE7429. First, a principal component analysis of the expression profiles of the GSE56814 and GSE56815 datasets was performed to reveal the gene expression data, as shown in Figures 2A,B. Second, we identified the optimal number of clusters by computing the CPI (blue line) and gap statistics (red line) in the cohort of osteoporosis patients after combining GSE56814 with GSE56815 by integrating the similarity network fusion and consensus clustering algorithm (SNF-CC). K = 3. Among them, when k = 3, the consensus matrix heatmap still maintains clear and clear boundaries, indicating that the clustering of samples is stable and robust, Figure 2C. We defined these three osteoporosis subtypes as C1, C2, and C3. Finally, we identified three osteoporosis subtypes by principal component analysis, as shown in Figure 2D.

### Differential gene expression profiles of osteoporosis subtypes

**FIGURE 3:**
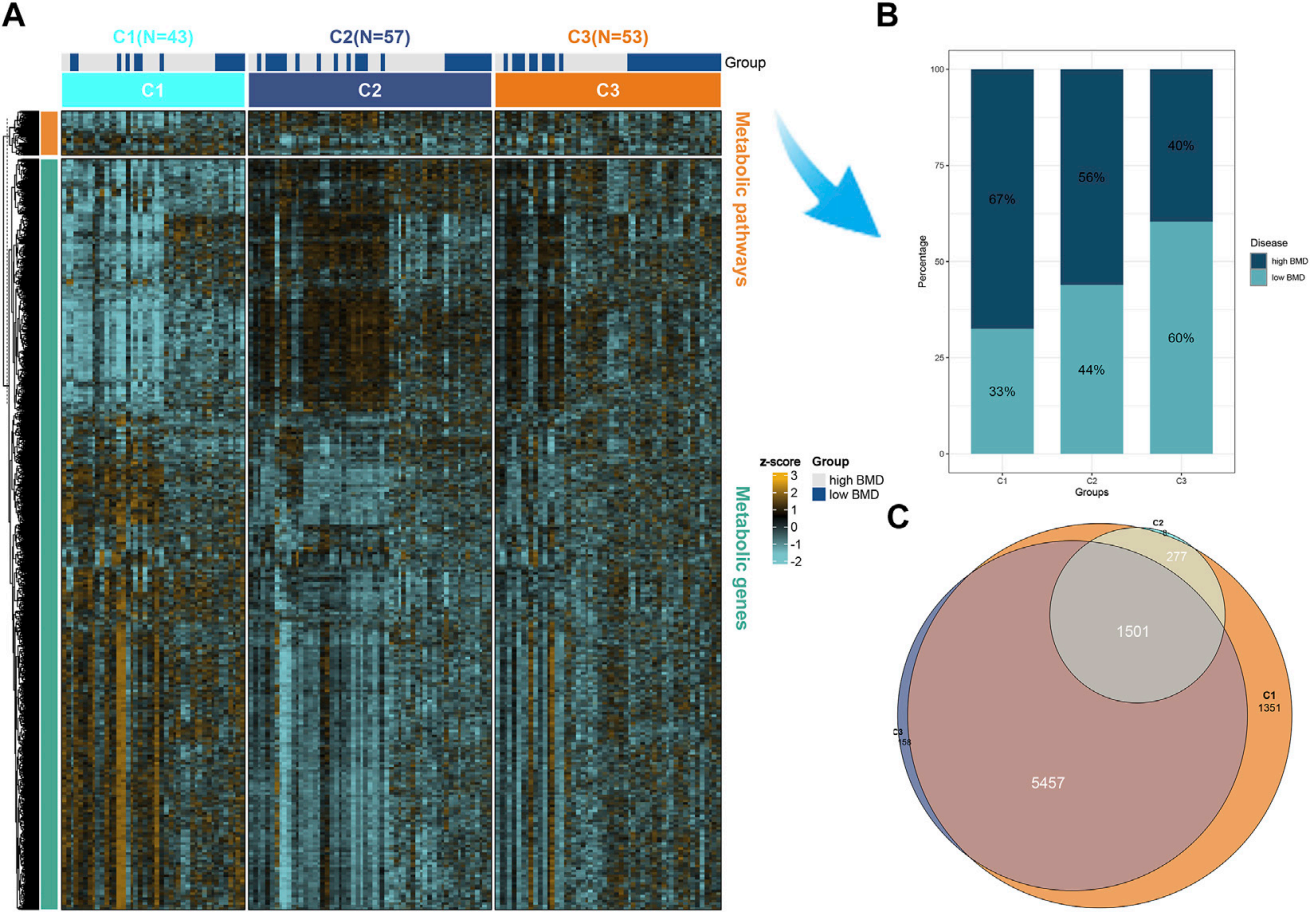
Representative genes for each subtype. **(A)** Heatmap of the expression of metabolic genes and metabolic pathways by BMD in the high and low groups in three subtypes of osteoporosis patients. **(B)** The proportion of BMD in high and low groups in three different subtypes of osteoporosis. **(C)** Differential genes were calculated for each of the two subgroups and intersected. Subtypes 1, 2, and 3 are 1,351, 8, and 158 representative genes, respectively. Multi-omics enrichment mapping of metabolic-related differential genes.

Additionally, we plotted metabolic pathways with metabolic gene heatmaps showing that the high BMD group clustered with the CI subtype and the low BMD group clustered with the C3 subtype. Figure 3A. We plotted the percentage bar graph of three subtypes between different BMDS. Among them, the C1 subtype accounted for a high proportion in patients with high BMD osteoporosis, the C3 subtype had a high proportion in patients with low BMD osteoporosis, and the C2 subtype had a similar proportion in patients with different BMD osteoporosis. As shown in Figure 3B, we used the R language limma package for gene difference analysis between subtypes and plotted Venn diagrams to identify representative genes for each subtype. Of these, 1,501 were shared among the three subtypes; see supplementary material 2. Meanwhile, 1,351, 8, and 158 differentially expressed genes were identified for different subtypes C1, C2, and C3, respectively (Figure 3C), and volcano plots were drawn to represent the gene signatures shown in supplementary materials 3, 4, and 5.

### Multi-omics enrichment mapping of metabolic-related differential genes

**FIGURE 4:**
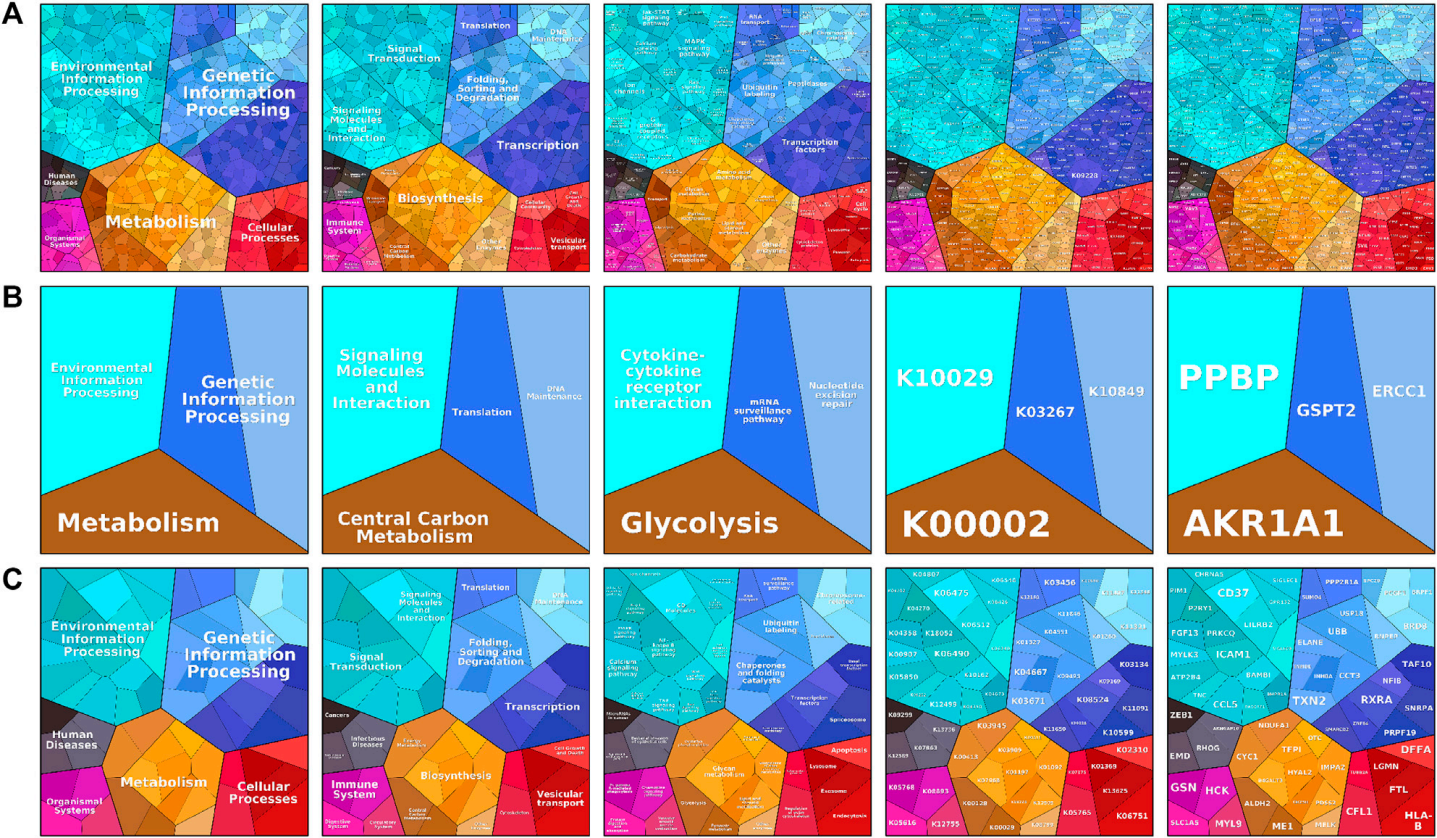
The proteomaps pathway analysis plot includes information process area polygon, disease area, pathway area, protein area, and gene area modules. **(A)** Indicates relevant information in osteoporosis type C1. **(B)** Denotes relevant information on osteoporosis type C2. **(C)** Indicates relevant information for osteoporosis type C3. Screening and differential expression of metabolic-related genes in osteoporosis.

To visualize the differences in enrichment information of the signature genes in different subgroups and to confirm the results obtained in the enrichment analysis using additional bioinformatics tools, we uploaded the signature genes of the three subgroups separately to the web-available interactive software proteomaps. In the created proteomap visualization rectangle, the whole region was divided into color-coded polygons representing the top categories, and the top category regions were subdivided into disease region subcategories, functionally related protein sharing common region subcategories, and related gene sharing common region subcategories, as shown in Figure 4. The C1 subtype was enriched in metabolism concerning enrichment in biosynthesis, amino acid metabolism, lipid, and steroid metabolism, with the main protein being K00718 and the main gene being FUT, as shown in Figure 4A. The C2 subtype was metabolically enriched in the central carbon metabolism, glycolysis pathway, k00002 protein, gene *AKR1A1,* Figure 4B. The C3 subtype was metabolically enriched in biosynthesis, glycan metabolism pathways of polysaccharides and the main proteins were k07968, k01197, and k03909, and the main genes were *B4GALT3, HYAL2,* and *TFPI,* as shown in Figure 4C.

### Screening and differential expression of metabolic-related genes in osteoporosis

**FIGURE 5:**
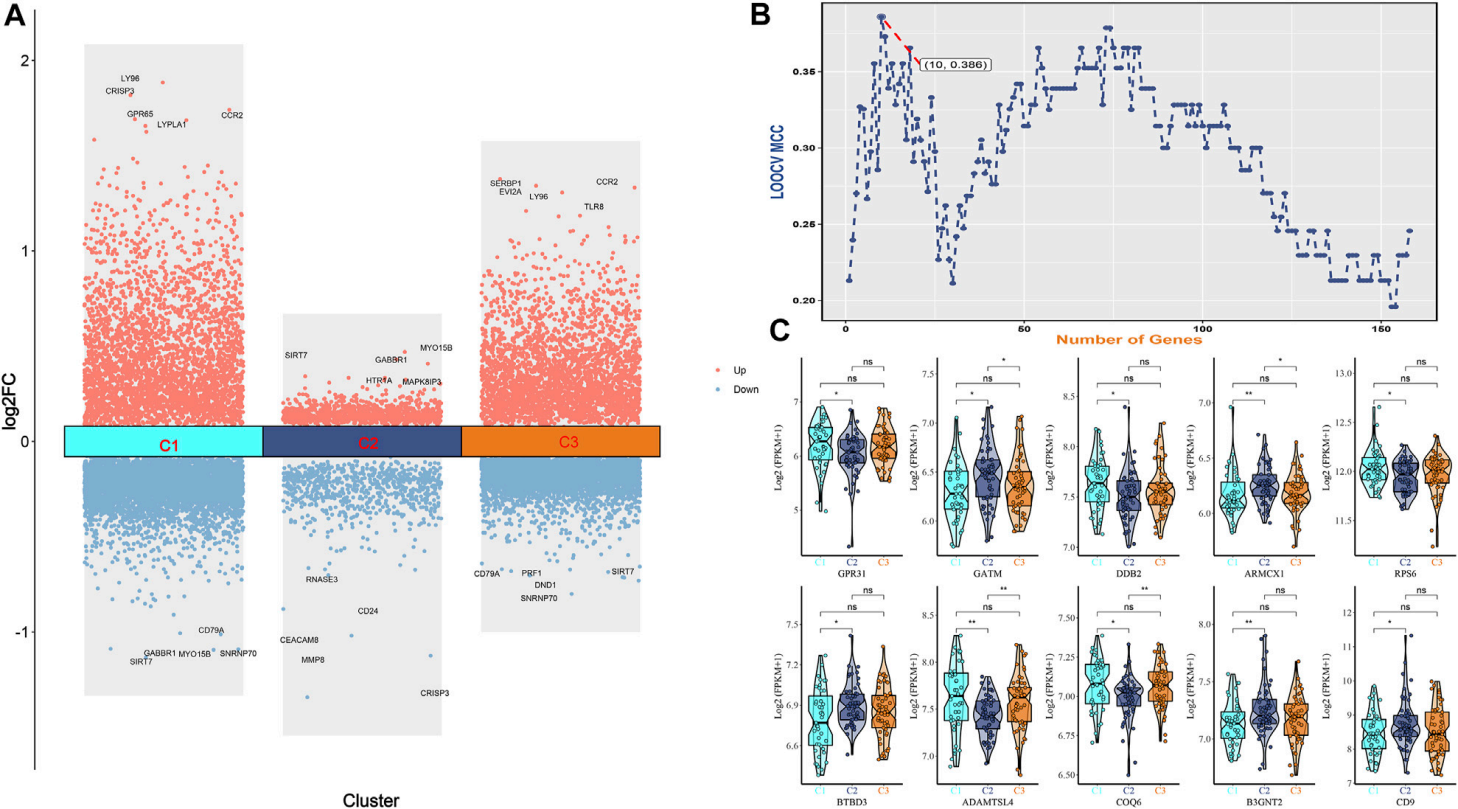
**(A)** Bar graph of differential genes of three osteoporosis subtypes. **(B)** IFS curves are based on the top 160 mrmr genes. The x-axis is the number of genes, and the y-axis is the prediction performance, that is, loocv MCC. When 10 genes were used, the peak MCC was 0.386. **(C)** The expression difference box plot represents the difference in expression levels of GPR31, GATM, DDB2, ARMCX1, RPS6, BTBD3, ADAMTSL4, COQ6, B3GNT2, and CD9 genes among the three isoforms.

We visualized the differential genes of different osteoporosis subtypes, as shown in Figure 5A. Based on the top 160 mRMR genes screened, we determined the optimal number of genes as feature genes by constructing 100 SVM classifiers and applying the incremental feature selection (IFS) method, as shown in Figure 5B. When the number of genes was 10, the peak MCC was 0.386, and these 10 genes were considered to be characteristic genes related to metabolism in osteoporosis, including *GPR31, GATM, DDB2, ARMCX1, RPS6, BTBD3, ADAMTSL4, COQ6, B3GNT2,* and *CD9*. Subsequently, we visualized violin plots based on the expression of 10 candidate genes in different subtypes of osteoporosis. *GPR31, DDB2, RPS6, ADAMTSL4,* and *COQ6* were expressed in C1 and C2 subtypes, C1>C2. *GATM, ARMCX1, BTBD3, B3GNT2,* and *CD9* were expressed in C1 and C2 subtypes, C1<C2. In the expression of *GATM* and *ARMCX1* in C2 and C3 subtypes, C2>C3. *ADAMTSL4* and *COQ6* were expressed in C2 and C3 subtypes, C2<C3. Interestingly, there was no statistical significance in comparing C1 with C3. (* means *p* = 0.05, ** means *p* = 0.01, *** means *p* = 0.001, ns means no statistical significance), as shown in Figure 5C.

### Signature gene expression data validation

**FIGURE 6:**
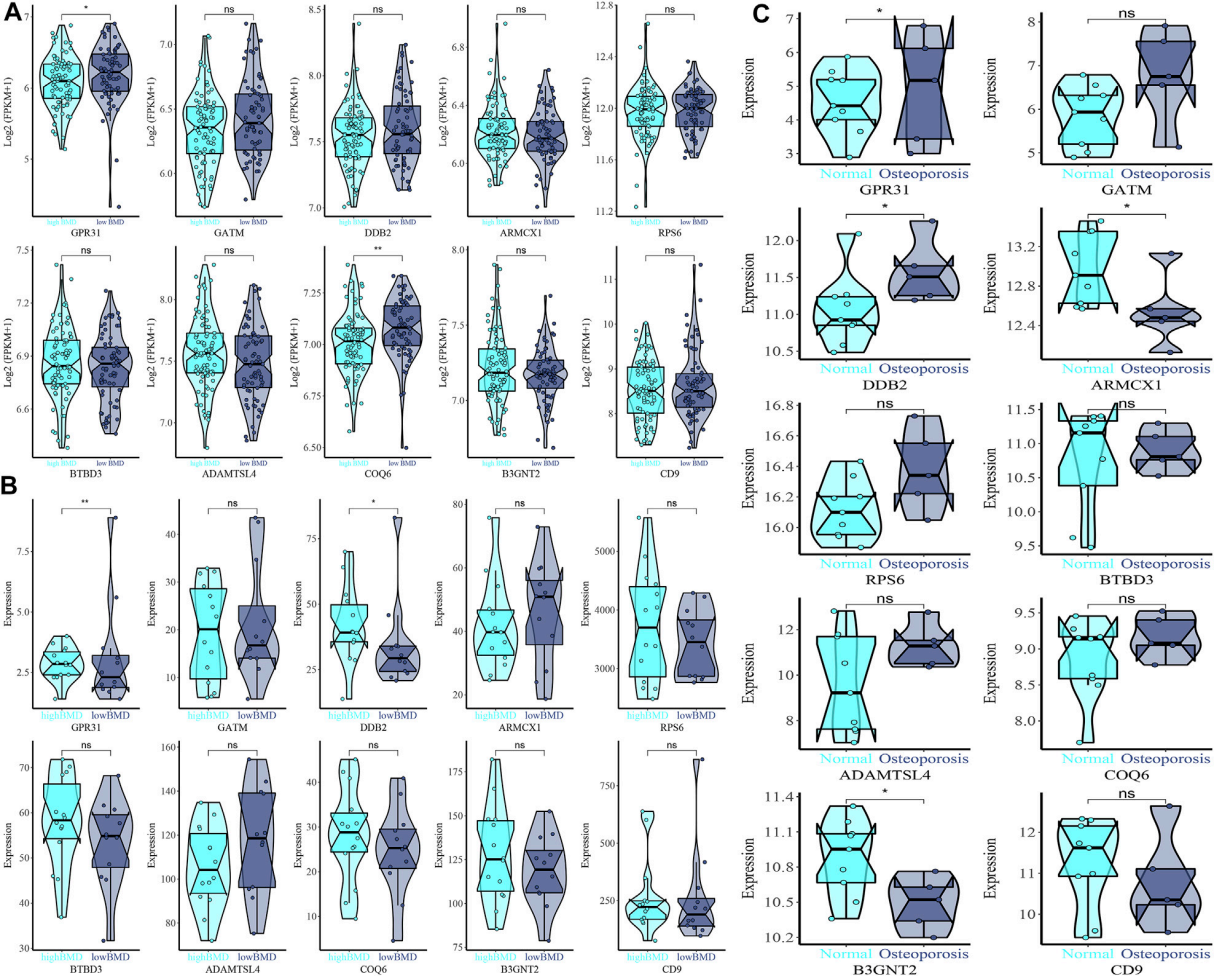
Expression difference violin plot of the difference in expression levels of GPR31, GATM, DDB2, ARMCX1, RPS6, BTBD3, ADAMTSL4, COQ6, B3GNT2, and CD9 genes in the external validation dataset. **(A)** GSE35959 and GSE7429 merged dataset validation. **(B)** GSE7429 dataset validation. **(C)** GSE35959 dataset validation.

Based on *GPR31, GATM, DDB2, ARMCX1, RPS6, BTBD3, ADAMTSL4, COQ6, B3GNT2,* and *CD9* gene expression in GSE35959 and GSE7429 datasets, we drew violin plots for data visualization for expression verification. Among them, in the expression of *GPR31* in the combined data, high BMD < low BMD, as shown in Figure 6A. *GPR31* is expressed in GSE7429 data, high BMD > low BMD, DDB2 is expressed in GSE7429 data, and high BMD > low BMD, as shown in Figure 6B. In GSE35959 data expression of *GPR31* and *DDB2*, normal group < osteoporosis group, while *ARMCX1* and *ADAMTSL4* in GSE35959 data expression, normal group > osteoporosis group, as shown in Figure 6C.

### Immune correlates analysis

**FIGURE 7:**
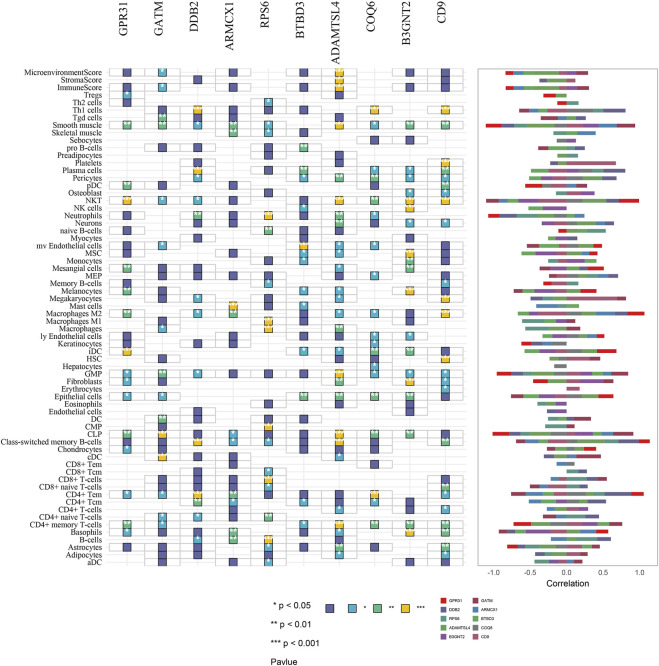
Correlation plot of immune and stromal cell composition based on GPR31, GATM, DDB2, ARMCX1, RPS6, BTBD3, ADAMTSL4, COQ6, B3GNT2, and CD9 gene analysis. * = *p* < 0.05, ** = *p* < 0.01, *** = *p* < 0.001.

Finally, we used xCell to infer *GPR31, GATM, DDB2, ARMCX1, RPS6, BTBD3, ADAMTSL4, COQ6, B3GNT2* and *CD9* genes for 64 immune and stromal cell types. These consist of 16, 15, 14, 10, 17, 13, 27, 19, and 24 representative cells, respectively, as shown on the correlation plot (Figure 8fig8). The representative cells of each gene mainly include NKT (Natural killer T-cells), iDC (Immature dendritic cells); CLP (Common lymphoid progenitors), cDC (Xonventional dendritic cells); Th1 cells (Type 1 T-helper cells), Plasma cells, CD4^+^ Tem (CD4^+^ effector memory T-cells), Class-switched memory B-cells; Mast cells, Macrophages M2; CD8^+^ T-cells, B-cells; mv Endothelial cells (Microvascular endothelial cells); Class-switched memory B-cells, NKT (Natural killer T-cells); Th1 cells (Type 1 T-helper cells), CD4^+^ Tem (CD4^+^ effector memory T-cells); Fibroblasts, Basophils, MSC (Mesenchymal stem cells); Platelets, Megakaryocytes, HSCs (Hematopoietic stem cells). As shown in Figure 7.

### Discussion

Osteoporosis is still a challenge to the public health problem (Compston et al., 2019). Meanwhile, the pathogenesis associated with osteoporosis, especially postmenopausal osteoporosis, still needs to be explored. Therefore, we conducted a multi-omics exploration of the mechanisms underlying the etiological heterogeneity of osteoporosis. We found that osteoporosis could be classified into three metabolic subtypes by cluster analysis and enrichment analysis of gene expression microarrays: lipid and steroid metabolic, glycolysis-related, and glycan. Meanwhile, we identified 10 potentially metabolically relevant signature genes by machine learning. In conclusion, our study provides new insights into osteoporosis as potential pathogenesis and gene as a therapeutic target in terms of metabolic relevance.

In our study, the 10 genes associated with osteoporosis metabolism were screened by machine learning, and these 10 genes were *GPR31, GATM, DDB2, ARMCX1, RPS6, BTBD3, ADAMTSL4, COQ6, B3GNT2,* and *CD9*. Meanwhile, in our external data validation, *GPR31* was highly expressed in patients with low-level bone mineral density and patients with osteoporosis. Regrettably, there are currently few studies on *GPR31*, and studies related to osteoporosis have not been reported. However, some studies suggest that *GPR31* is a target receptor for 12(S)-hydroxyeicosatetraenoic acid (12(S)-HETE) and has the same lipid receptor properties as other proton-sensing GPCRs (Guo et al., 2011). *GPR31* has the highest protein identity (up to 36%) to hydroxy carboxylate receptor (HCAR) 1-3 and is activated under acidic conditions (Mashiko et al., 2019). Based on the above findings, we can infer that *GPR31* as a target receptor is involved in osteoporosis by mediating the imbalance of lipid metabolism through the regulation of 12(S)-HETE.

In the results of this study, osteoporosis subtype 1 was mainly related to lipid and steroid metabolism, and the main proteins and genes were K00718 and FUT. There is reported *in vitro* evidence that lipids are involved in the development of osteoporosis (Parhami et al., 2001). However, the association between lipid metabolism and osteoporosis in human studies remains controversial. Numerous studies show an inverse correlation between lipid biomarkers and bone mineral density (BMD). Ersoy and Lahon et al. found a positive correlation between low-density lipoprotein cholesterol (LDL-C) and bone density (Lahon et al., 2016; Ersoy et al., 2017). Interestingly, Ghadiri-Anari’s study showed no association between lipids and bone density (Ghadiri-Anari et al., 2016). Similarly, in a cross-sectional study by Kan et al., higher total cholesterol and triglycerides were strongly associated with higher osteoporosis risk in humans (Kan et al., 2021). Studies have also shown that high-density lipoprotein cholesterol (HDL-C) is elevated in patients with osteoporosis (Zhao et al., 2021b). However, studies on the relationship between the protein K00718 and the FUT gene and osteoporosis have not been reported. However, based on the findings, we infer that osteoporosis subtype 1 may be involved in lipid and steroid metabolism.

Interestingly, osteoporosis subtype 2 is metabolically associated with the glycolytic metabolic pathway, including the protein K00002 and the AKR1A1 gene. It is well known that osteoclast-mediated bone resorption leads to osteoporosis more than osteoblast-mediated bone formation, leading to an imbalance in bone homeostasis. There is increasing evidence that glycolysis in cells of the osteoblastic lineage is directly stimulated by various bone anabolic signals. Recent studies suggest that parathyroid hormone (PTH) can stimulate aerobic glycolysis to promote bone anabolism through IGF signaling (Esen et al., 2015). Meanwhile, WntSignals directly regulate cellular metabolism by stimulating aerobic glycolysis, glutamine catabolism, and fatty acid oxidation in osteoblastic lineage cells (Karner and Long, 2017). In particular, experiments showed that the Wnt3a-Lrp5 signaling pathway could increase Glut1, Hk2, Ldha, and Pdk1 downstream of mTORC2 and Akt activation while inducing osteoblastic differentiation of the bone marrow mesenchymal progenitor cell line ST2 (Esen et al., 2013).

On the other hand, increased glycolysis, oxidative phosphorylation, and lactate production were found during nuclear factor kappa B ligand (RANKL)-induced osteoclast differentiation from mouse bone marrow macrophages. Furthermore, osteoclastogenesis can be delayed by blocking ATP production with a mitochondrial complex inhibitor or the ATP synthase inhibitor oligomycin, a finding that suggests that osteoclastogenesis may be regulated by changes in the concentration of metabolic substrates (Kim et al., 2007). Recently, experimentally reducing the rate of glycolysis with galactose impairs collagen I degradation, while inhibition of mitochondrial complex I with non-cytotoxic doses of rotenone enhances osteoclast activity, suggesting that human osteoclasts are sugar-dependent bone resorption by glycolysis (Lemma et al., 2016). However, more research is needed to elucidate the full mechanism of glycolysis between osteoblasts and osteoclasts. These comprehensive insights into osteoblast and osteoclast metabolism and its regulation may reveal molecular targets for osteoporosis.

More importantly, osteoporosis subtype 3 is metabolically enriched in biosynthesis and polysaccharide metabolism pathways. The main proteins are K07968, K01197, and K03909, and the main genes are *B4GALT3, HYAL2,* and *TFPI*. The association of these genes and proteins with osteoporosis has not been reported. However, studies show that polysaccharides have therapeutic effects on several types of osteoporosis, including postmenopausal osteoporosis, senile osteoporosis, and glucocorticoid-induced secondary osteoporosis, by promoting osteoblast differentiation and activity and reducing osteoclast differentiation and activity (Lei et al., 2021). It has also been shown that Wnt/β-catenin signaling pathways play an important role in BMSC self-renewal, directional differentiation, osteoblast proliferation and formation, and apoptosis. Li et al. showed that BMP9 is a direct target of miR-152 and that aspartate downregulates the expression of miR-152 and promotes upregulation of BMP9, which activates PI3K/Akt and Wnt in BMSCs/β-Catenin signaling pathway (Li et al., 2019). Wnt3a and Wnt10b expression in MSCs can activate Wnt in a process that promotes osteoblast differentiation/β-catenin signaling pathway to promote proliferation of BMSCs (Liu et al., 2008). Meanwhile, DKK-1 is a suppressor of Wnt signaling and can inhibit bone formation leading to osteoporosis, and antagonizing DKK-1 can increase bone mass (Anastasilakis et al., 2010). Research suggests that polysaccharide polysaccharide (RPP) can promote osteoblast differentiation and mineralization by regulating ERK/GSK-3β/β-catenin signaling pathway (Peng et al., 2018). Meanwhile, astragalus polysaccharide (ASP) can effectively alleviate oxidative stress-mediated osteoporosis in ovariectomized rats by regulating the FoxO3a/Wnt2/β-catenin pathway (Ou et al., 2019). On the other hand, Lycium barbarum polysaccharide (LYP)-mediated serum can activate the expression of Wnt signaling pathway-related proteins β-catenin and Wnt10b and promote the differentiation and mineralization of bone marrow mesenchymal stem cells into osteoblasts (Wang et al., 2017). Huang et al. found that two novel polysaccharides, CBP70-1-1 and CBP70-1-2, promoted osteoblast proliferation at low concentrations (Huang et al., 2020a). Chen et al. found that Gastrodia elata Blume polysaccharide (wss25) could inhibit the expression of the BMP2/Smad1 pathway and osteoclast differentiation (Chen et al., 2015). Gracilaria lemaneiformis polysaccharide (APP) can upregulate mir-70 expression and modulate Wnt/β-catenin signaling, enhancing alkaline phosphatase activity and promoting osteoblast proliferation and differentiation, and increasing osteoblast differentiation marker proteins BMP2, Runx2, osterix, and osteocalcin (Huang et al., 2020b; Huang et al., 2020c). In summary, there is a close relationship between polysaccharide metabolism and the development of osteoporosis, so we speculate that the proteins k07968, k01197, k03909, and genes *B4GALT3, HYAL2, and TFPI* may have relevance in the regulation of multiple signaling pathways by polysaccharide metabolism in the development of osteoporosis.

To clarify the interaction of immune cells and stromal cells in osteoporosis, we analyzed the correlation of osteoporosis metabolism-related genes in 64 immune and stromal cell types. The results suggest that a variety of immune cells and stromal cells may be relevant in osteoporosis. Studies have suggested that B and T cells are important stabilizers of bone turnover and key regulators of peak bone mass *in vivo* to maintain bone homeostasis and achieve peak bone mass *in vivo* (Li et al., 2007). On the other hand, it is believed that under conditions such as estrogen deficiency, infection, and inflammation, T-cell-dependent release of IFN- γ Can inhibit IFN- γ Signaling and osteoclast formation, in turn, prevent bone loss (Gao et al., 2007). In contrast, dendritic cells can release RANKL to initiate osteoclastogenesis and bone resorption in response to inflammatory osteoporosis (Pietschmann et al., 2016). At the same time, dendritic cells, similar to B lymphocytes, may participate in the regulation of physiological bone remodeling by secreting receptor activator of nuclear factor kappa-B ligand receptor (RANKL)-induced receptor osteoprotegerin (OPG), thereby inhibiting osteoclast activation (Walsh and Choi, 2014). A variety of immune cells are involved in bone anabolic processes.

As a systemic skeletal system disease, osteoporosis must find more ways to unravel pathogenesis research. Metabolic abnormalities have entered osteoporosis as a new perspective and may become a new direction for research on new biomarkers for osteoporosis. Therefore, based on the correlation analysis of multi-omics and metabolic-related genes and osteoporosis, this study explored the characteristic genes that can guide deeper osteoporosis research. Regrettably, our study still has certain limitations. First, using xcell to represent the correlation of immune to stromal cell abundance with direct measurements is not the same, and inferences as abundance scores cannot be interpreted as true proportions. Therefore, we cannot guarantee the reliability of our findings in xcell’s results and need to be treated with caution. Meanwhile, our genotyping of osteoporosis and signature genes by integrating multi-omics data needs more clinical samples and molecular cell experiments for validation, but our study still has certain guiding significance.

## Conclusion

Based on the clustering analysis of gene expression in patients with osteoporosis and machine learning, we identified different metabolism-related subtypes and characteristic genes of osteoporosis, which will help to provide new ideas for the metabolism-related pathogenesis of osteoporosis and provide a new direction for follow-up research.

## Data Availability

The original contributions presented in the study are included in the article/Supplementary Material, further inquiries can be directed to the corresponding authors.
